# Neuropathology of brain and spinal malformations in a case of monosomy 1p36

**DOI:** 10.1186/2051-5960-1-45

**Published:** 2013-08-02

**Authors:** Naoko Shiba, Ray AM Daza, Lisa G Shaffer, A James Barkovich, William B Dobyns, Robert F Hevner

**Affiliations:** 1 Seattle Children’s Research Institute, 1900 Ninth Ave., Box C9S-10, Seattle, WA 98101, USA; 2 Signature Genomic Laboratories, Spokane, WA, USA; 3 Department of Radiology, University of California, San Francisco, San Francisco, CA, USA; 4 Department of Pediatrics, University of Washington, Seattle, WA, USA; 5 Department of Neurological Surgery, University of Washington, Seattle, WA, USA

**Keywords:** Periventricular nodular heterotopia, Hippocampal malrotation, Cortical dysgenesis, Malformations of cortical development, Hydromyelia, Mental retardation, Epilepsy

## Abstract

Monosomy 1p36 is the most common subtelomeric chromosomal deletion linked to mental retardation and seizures. Neuroimaging studies suggest that monosomy 1p36 is associated with brain malformations including polymicrogyria and nodular heterotopia, but the histopathology of these lesions is unknown. Here we present postmortem neuropathological findings from a 10 year-old girl with monosomy 1p36, who died of respiratory complications. The findings included micrencephaly, periventricular nodular heterotopia in occipitotemporal lobes, cortical dysgenesis resembling polymicrogyria in dorsolateral frontal lobes, hippocampal malrotation, callosal hypoplasia, superiorly rotated cerebellum with small vermis, and lumbosacral hydromyelia. The abnormal cortex exhibited “festooned” (undulating) supragranular layers, but no significant fusion of the molecular layer. Deletion mapping demonstrated single copy loss of a contiguous 1p36 terminal region encompassing many important neurodevelopmental genes, among them four *HES* genes implicated in regulating neural stem cell differentiation, and *TP73*, a monoallelically expressed gene. Our results suggest that brain and spinal malformations in monosomy 1p36 may be more extensive than previously recognized, and may depend on the parental origin of deleted genes. More broadly, our results suggest that specific genetic disorders may cause distinct forms of cortical dysgenesis.

## Background

Monosomy 1p36 is the most common subtelomeric chromosomal deletion syndrome, with an estimated incidence of 1/5000-1/10000 births [1]. Of the ~95% of deletions that arise *de novo*, 60% affect the maternally derived chromosome [1], and manifest as various kinds of chromosomal rearrangements (e.g., interstitial deletions, derivative chromosomes, and more complex rearrangements, as well as pure terminal deletions). The clinical phenotype of monosomy 1p36 includes mental retardation, developmental delay, muscular hypotonia, speech delay, epilepsy, behavioral disorders (including panic, aggressiveness, and self-injury), sensorineural hearing loss, eye/vision problems, congenital heart defects, cardiomyopathy, gastrointestinal problems, precocious puberty, hyperphagia/obesity, and dysmorphic features (deep-set eyes, flat nasal bridge, pointed chin, large and late-closing anterior fontanel, and clinodactyly) [1-5]. Mental retardation and developmental delay are seen in nearly 100% of patients, and are usually moderate or severe [5,6]. Generalized hypotonia is observed in >90% of patients, and epilepsy in 50-80% [4,6].

The chromosome deletion in most cases maps to the distal 10.5 Mb of 1p36, although the size varies and there is no common break point [1]. There is little correlation between the deletion size and the *number of clinical features*; even relatively small deletions (<3 Mb) can present with most of the typical phenotypic features [3,7]. Indeed, it has been suggested that some features of monosomy 1p36 might result from positional effects rather than a simple contiguous gene deletion [8]. On the other hand, the *severity of neurological deficits* including epilepsy, mental retardation and sensorineural hearing loss, as well as congenital heart diseases, has been suggested to correlate with the deletion size [6,8]. Furthermore, some specific phenotypes in the monosomy 1p36 syndrome — including polymicrogyria (PMG), hyperphagia/obesity, mental retardation, seizures, hearing loss, and periventricular nodular heterotopia (PNH) — have been linked to critical regions within 1p36, each of which nevertheless contains multiple genes [2,4,9-11].

Although neuropsychiatric abnormalities are pivotal phenotypes in monosomy 1p36, their pathogenesis remains poorly understood. In most cases, neuroimaging reveals only mild or nonspecific changes such as microcephaly, ventriculomegaly, cerebral atrophy, abnormal patchy signal in white matter, and delayed myelination. Recently, however, evidence has accumulated that some fraction of monosomy 1p36 cases are affected with significant developmental brain malformations, including PMG, PNH, hypoplasia or agenesis of the corpus callosum, holoprosencephaly, cerebellar hypoplasia, and choroid plexus hyperplasia [10,12-19]. In one series, for example, 13 of 64 (20.3%) patients with monosomy 1p36 showed neuroimaging evidence of PMG [10].

In the present case report, we present the first neuropathological analysis of brain and spinal cord malformations in monosomy 1p36, from postmortem examination of a girl who died at 10 years of age. Our findings reveal new details of the malformations, and suggest that PMG in these patients represents a distinct form of cortical dysgenesis.

## Case presentation

### Clinical history

The subject was delivered at 39 weeks of gestation by caesarean section for breech position, to a 22 year-old, gravida 2, para 1 (1 spontaneous abortion) mother who had received special education in school. The father was a nonconsanguineous 37 year-old man with no known medical problems or significant family history. Birth weight was 2035 g (−3.7 SD). Apgar scores were 7 at 1 minute and 8 at 5 minutes. Neonatal cardiac evaluation showed a patent ductus arteriosus (PDA) and small ventricular septal defect (VSD). The PDA was ligated at age 7 weeks and the VSD closed spontaneously. She had severe conjugated hyperbilirubinemia transiently during early infancy, along with other findings suggestive of possible Alagille syndrome (posterior embryotoxon and butterfly vertebrae). However, liver biopsy showed no paucity of bile ducts (as present in Alagille syndrome) nor any other disease specific finding. Postnatal growth retardation (height < 5th centile), microcephaly (head circumstance < 5th centile), feeding difficulties, muscular hypotonia, and delayed motor and mental development were also noted. Infantile apneic spells were noted, prompting magnetic resonance imaging (MRI) that revealed cervicomedullary compression at the skull base; this was surgically decompressed. At 8 months of age, the patient was referred to the Department of Medical Genetics and was noted to exhibit dysmorphic features including bilateral low set small malformed ears, prominent forehead, deep set eyes, flat nasal bridge, pointed chin, short neck, and bilateral single transverse creases.

At age 13 months, growth hormone deficiency of unknown etiology was diagnosed and treated with growth hormone replacement therapy. The patient had severe gastro-esophageal reflux causing recurrent aspiration pneumonia; Nissen fundoplication with gastrostomy was done at 15 months of age. Precocious secondary sexual characteristics and clitoromegaly were noted at 22 months. The first apparent generalized tonic-clonic seizure occurred at age 5 years; seizures progressed, and were ultimately treated with combination anti-epileptic drug therapy. On serial neurological examinations, the patient never spoke any words nor walked, and her development remained profoundly delayed. She also had myopia with astigmatism and sensorineural hearing loss bilaterally. Self-injurious behavior (arm chewing) and teeth grinding were observed.

G-band karyotyping of peripheral blood lymphocytes showed a terminal deletion of chromosome 1p, read as 46,XX,del(1)(p36.31). The deletion in this region was confirmed by fluorescence in situ hybridization (FISH) using a subtelomeric DNA probe specific for the region, 1p36.3 (D1Z2; Oncor, Inc., cat #p5000-DG). The mother’s karyotype was normal; the father’s karyotype was not available.

**Figure 1:**
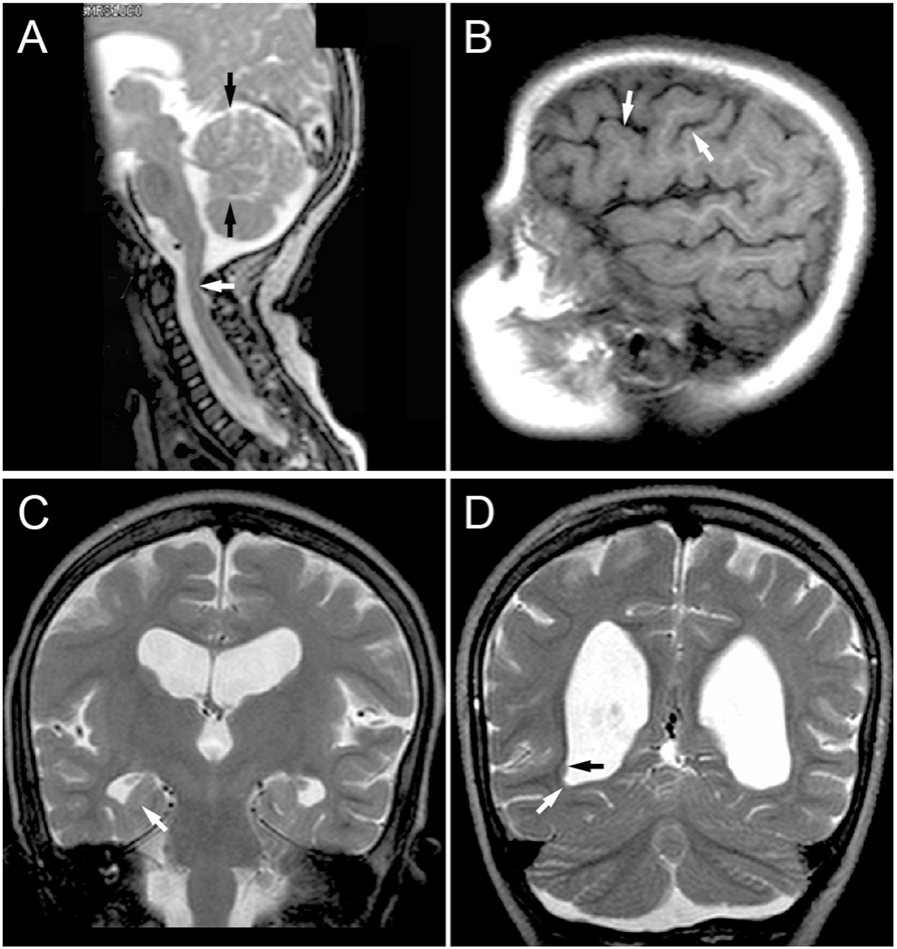
**MRI images at ages 1 and 10 years. (A)** Sagittal T2 weighted image at age 1 year showed a small cerebellar vermis (black arrows) and abnormal posterior arch of C1 (white arrow) causing compression of the posterior aspect of the upper cervical spinal cord. **(B)** Parasagittal T1 weighted image at age 10 years showed subtle irregularity of the cortical-white matter junction and the surface of the cortex in the left frontal lobe (arrows), consistent with polymicrogyria or similar dysgenesis. **(C)** Coronal T2 weighted image at age 10 years showed ventriculomegaly and malrotated hippocampi. **(D)** Coronal T2 weighted image at age 10 years at the level of the ventricular trigones showed periventricular nodular heterotopia of moderate (black arrow) and small (white arrow) sizes.

MRI examinations of the brain at several ages disclosed bilateral ventriculomegaly (Figure 1A-D). No progression of ventriculomegaly was noted upon repeated brain MRI. Electroencephalogram recordings showed slow background activity and frequent high amplitude multifocal discharges from the bilateral occipital lobes, compatible with focal epilepsy.

The patient died at age 10 years from cardiorespiratory failure.

### General autopsy findings

A complete autopsy was performed with familial consent. The postmortem interval was 23.5 hours. The lungs showed patchy severe pneumonia, with postmortem lung cultures positive for *Pseudomonas aeruginosa*. The heart showed dysplasia of atrioventricular valves, but no cardiomyopathy. No hepatic or renal abnormalities were present.

## Materials and methods

### Neuropathological analysis

The brain was removed within 48 hours after death and was fixed for two weeks in 10% formalin. Representative samples of formalin-fixed brain and spinal cord were embedded in paraffin, sectioned at 6 μm, and mounted on glass slides. Hematoxylin and eosin (H-E), Luxol fast blue (LFB), and cresyl violet (Nissl) stains utilized standard protocols. Age-matched normal human brain specimens were studied as controls.

Single-label immunoperoxidase staining was done using standard procedures. Double-label immunofluorescence was done as described [20]. The following primary antibodies were used: rabbit polyclonal antibodies against glial fibrillary acidic protein (GFAP; DAKO, 1:2000); mouse monoclonal antibodies against neurofilament heavy chain (clone N52; Chemicon, Temecula, CA; diluted 1:300); microtubule-associated protein 1B (MAP1B; Chemicon; 1:400), microtubule-associated protein 2 (MAP2; Chemicon; 1:500), synaptophysin (Chemicon; 1:400), rabbit polyclonal antibodies against calretinin (CR; Swant, Bellinzona, Switzerland; 1:2000). Primary antibody specificity was verified in control cortex. As a control for nonspecific binding of secondary antibodies, primary antibodies were omitted from some incubations.

Brightfield and fluorescence images were acquired using a Carl Zeiss Axio Imager Z1 microscope equipped with motorized stage and MosaiX software (Zeiss, USA). Images were photographed digitally and processed using Photoshop (Adobe, San Jose, CA, USA) to optimize brightness, contrast, and resolution. To map the positions of cells associated with deep and superficial cortical layers, N52+ cells and CR + cells (respectively) were plotted using different color dots (N52+ cells green, CR + cells red) against a black background, as described [21].

### High-resolution chromosomal deletion mapping

Chromosomal deletions and duplications were analyzed using a bacterial artificial chromosome (BAC)-based microarray (SignatureChip v3.0, Signature Genomic Laboratories, Spokane, WA) as described previously [22].

## Results

### Neuropathological findings

#### Macroscopic observations

**Figure 2:**
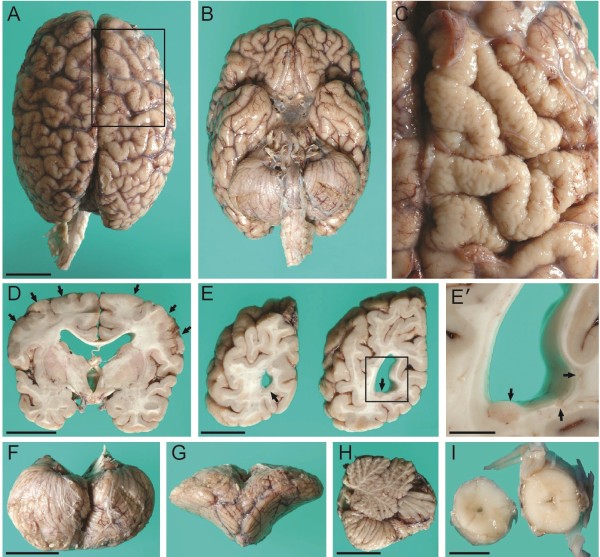
**Macroscopic neuropathology. (A)** Superior aspect of brain showed “Moroccan leather” appearance of frontal cortex bilaterally. **(B)** Inferior aspect of brain showed smooth gyral surfaces. **(C)** Surface of right frontal cortex corresponding to boxed area in **(A)**, with leptomeninges removed, showed irregular surface of cortical gyri. **(D)** Coronal slice through the hemispheres revealed bilateral polymicrogyria-like cortical dysgenesis (arrows) involving superior and middle frontal gyri. The gray-white matter junction was irregular in these regions. The corpus callosum was thin, and the lateral and third ventricles were enlarged. **(E)** Coronal slices of the left occipital lobe demonstrated multiple periventricular nodular heterotopia (arrows). **(E’)** Magnified view of boxed area in E with multiple heterotopia (arrows). **(F**-**H)** The cerebellum exhibited a small, anteriorly rotated vermis with normal number of lobules. **(I)** Central canal of the lower thoracic and upper lumbar cord was mildly dilated. Scale bars: (in A) A, B, 3 cm; C, 1 cm; D, 3 cm; E, 2 cm; E’, 1 cm; (in F) F, G, 3 cm; H, 2 cm; I, 0.5 cm.

The brain was small for age, weighing 858 g after formalin fixation, much less than expected (1290 g). The most prominent external brain abnormality was the “Moroccan leather”-like appearance of gyri in the dorsolateral frontal lobes bilaterally (Figure 2A-C). The gyral surface abnormalities, while most severe in the posterior portions of middle and superior frontal gyri, were not sharply delimited, but extended into adjacent cortical regions with gradually decreasing severity. Coronal slices of the hemispheres revealed bilateral ventriculomegaly, thin corpus callosum, focal cortical thickening especially in frontal lobes, and overall paucity of white matter (Figure 2D, E). Measurements of (non-polymicrogyric) cortex at several sulcal fundi showed the thickness ranged from 4.5 -7.6 mm, whereas normal cortex has a thickness of 3-4 mm [23]. Multiple periventricular heterotopias were identified, exhibiting both thin laminar and discrete nodular morphologies, scattered in the walls of the ventricular atria as well as occipital and temporal horns bilaterally (Figure 2E, E’). The macroscopic PNH measured ~0.3–1.0 cm in greatest dimension. The hippocampi were malrotated bilaterally (see below). The cerebellum exhibited a displaced, small, and anteriorly rotated vermis with normal organization of lobules (Figure 2F-H). The central canal of the lower thoracic and upper lumbar cord was mildly dilated (hydromyelia) (Figure 2I).

#### Histopathology of PMG-like cortical dysgenesis

**Figure 3:**
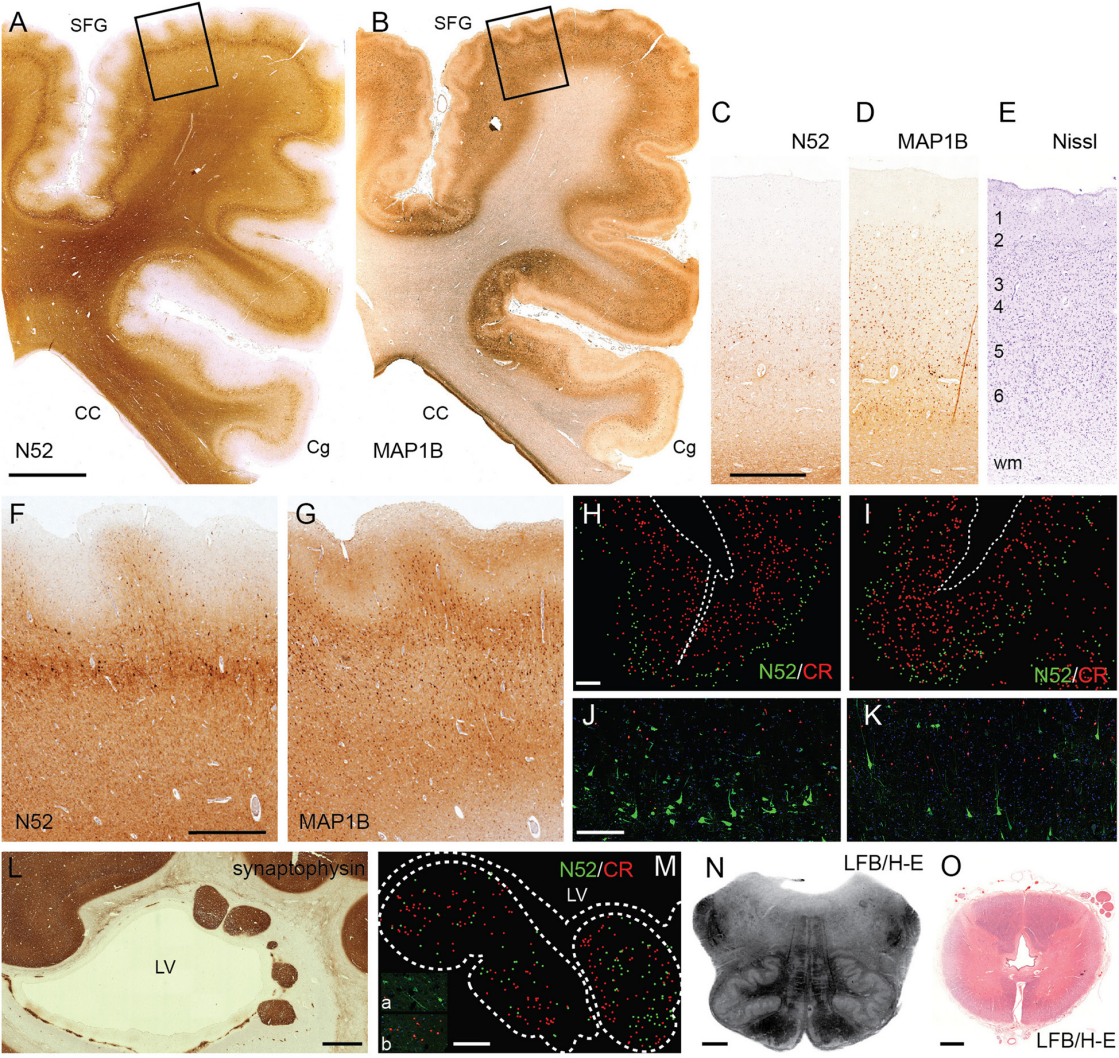
**Histologic neuropathology. (A**, **B)** Polymicrogyria-like cortical dysgenesis stained by immunohistochemistry for **(A)** N52 and **(B)** MAP1B. Note “festooned” supragranular layers in the patient’s superior frontal gyrus (SFG) and adjacent dorsolateral frontal cortex (superior up, lateral left). The cingulate cortex (Cg) was unaffected, except in the depth of the cingulate sulcus. The corpus callosum (CC) was hypoplastic. **(C**-**E)** Lamination of normal, age-matched control cortex demonstrated by **(C)** N52, **(D)** MAP1B, and **(E)** cresyl violet (Nissl) stains. **(F**, **G)** Polymicrogyria -like cortical dysgenesis seen at higher magnification (boxed areas from A, B). **(H**-**K)** Two-color, double-label immunofluorescence detection of N52 (green) and calretinin (red) in control **(H**, **J)** and patient **(I**, **K)** cerebral cortex. Low magnification views **(H**, **I)** show dot plots of immunolabeled cells. In patient cortex, the number and size of N52+ neurons appeared smaller, but the relative positions of N52+ and calretinin + neurons were unaltered. **(L)** Multiple synaptophysin-immunoreactive periventricular nodular heterotopia and small irregular heterotopia were identified in occipitotemporal regions adjacent to the lateral ventricle (LV). **(M)** Periventricular heterotopia exhibited N52 (green) and calretinin (red) immunoreactivity, represented in dot plots. Insets (a, b) show higher magnification photomicrographs. **(N)** The inferior olivary nuclei appeared slightly simplified with reduced convolutions. **(O)** The central canal of the spinal cord showed mild hydromyelia with a cross-shaped dilatation in lower thoracic and upper lumbar levels. Note also pallor of anterior and lateral funiculi. Scale bars: (in **A**) **A**, **B**, 5 mm; (in **C**) **C**-**E**, 1 mm; (in **F**) **F**, **G**, 1 mm; (in **H**) **H**, **I**, 500 μm; (in **J**) **J**, **K**, 250 μm; **L**, 2 mm; **M**, 500 μm; **N**, 2 mm; **O**, 2 mm.

Routine histological stains (H-E, LFB, Nissl) demonstrated six-layered cortex with possible “festooning” of the supragranular layers, but little or no significant fusion of the molecular layer. Rather, the molecular layer appeared to have variable thickness and irregular contours. To further analyze the PMG-like cortex, we used immunohistochemistry to study layer-specific antigens [24], including: N52 (neurofilament heavy chains, phosphorylated and non-phosphorylated), labeling pyramidal neurons in layers 5 and 3; MAP1B, labeling deep more intensely than in superficial layers; and CR, labeling interneurons in layers 2–3 [24]. These studies revealed additional details of festooning and variable thickness in supragranular layers (Figure 3A-K). First, it was clear that macroscopic PMG-like bumps on the cortical surface (Figure 2C) correlated with thickening or convex undulations of the supragranular layers, while tiny sulci correlated with supragranular thinning or concave undulations. Furthermore, the immunostains revealed that PMG-like cortical dysgenesis was more widespread than appreciated from abnormal surface morphology. For example, foci of supragranular festooning were evident not only in dorsolateral regions, but also in medial frontal and cingulate cortex, especially at the depths of sulci (Figure 3A, B). Nevertheless, the relative positions of supragranular and infragranular layers were preserved, as revealed by double labeling to detect CR (layers 2–3) and N52 (layers 5 and 3) in the same sections (Figure 3H-K). The cerebral white matter was atrophic, gliotic, and hypomyelinated (not shown).

#### Histopathology of periventricular heterotopia

The neuronal identity of PNH and adjacent thin laminar heterotopia along the walls of the occipital and temporal horns was confirmed by immunohistochemistry to detect synaptophysin (Figure 3L). In addition, many numerous smaller nodular or linear heterotopia (<3 mm), often aligned along the interface between the subventricular zone and white matter, were synaptophysin immunoreactive (Figure 3L). These findings indicated that heterotopia, like regions of cortical dysgenesis, were more extensive than appreciated from macroscopic examination. Also, the PNH contained multiple neuron types, including N52+ pyramidal neurons as well as CR + interneurons (Figure 3M). Interestingly, in some PNHs, N52+ neurons were located predominantly in the center of the nodule, and CR + interneurons around the outside (Figure 3M, nodule at right). This observation suggests that some nodules may have had a rudimentary laminar structure.

#### Histopathology of the brainstem, cerebellum, and spinal cord

The inferior olivary nuclei appeared mildly hypoconvoluted (Figure 3N). The cerebellar cortex exhibited patchy loss of Purkinje neurons; the dentate nuclei were unremarkable. The spinal central canal was moderately dilated at lower thoracic and upper lumbar levels (Figure 3O).

#### Histopathology of the hippocampus

**Figure 4:**
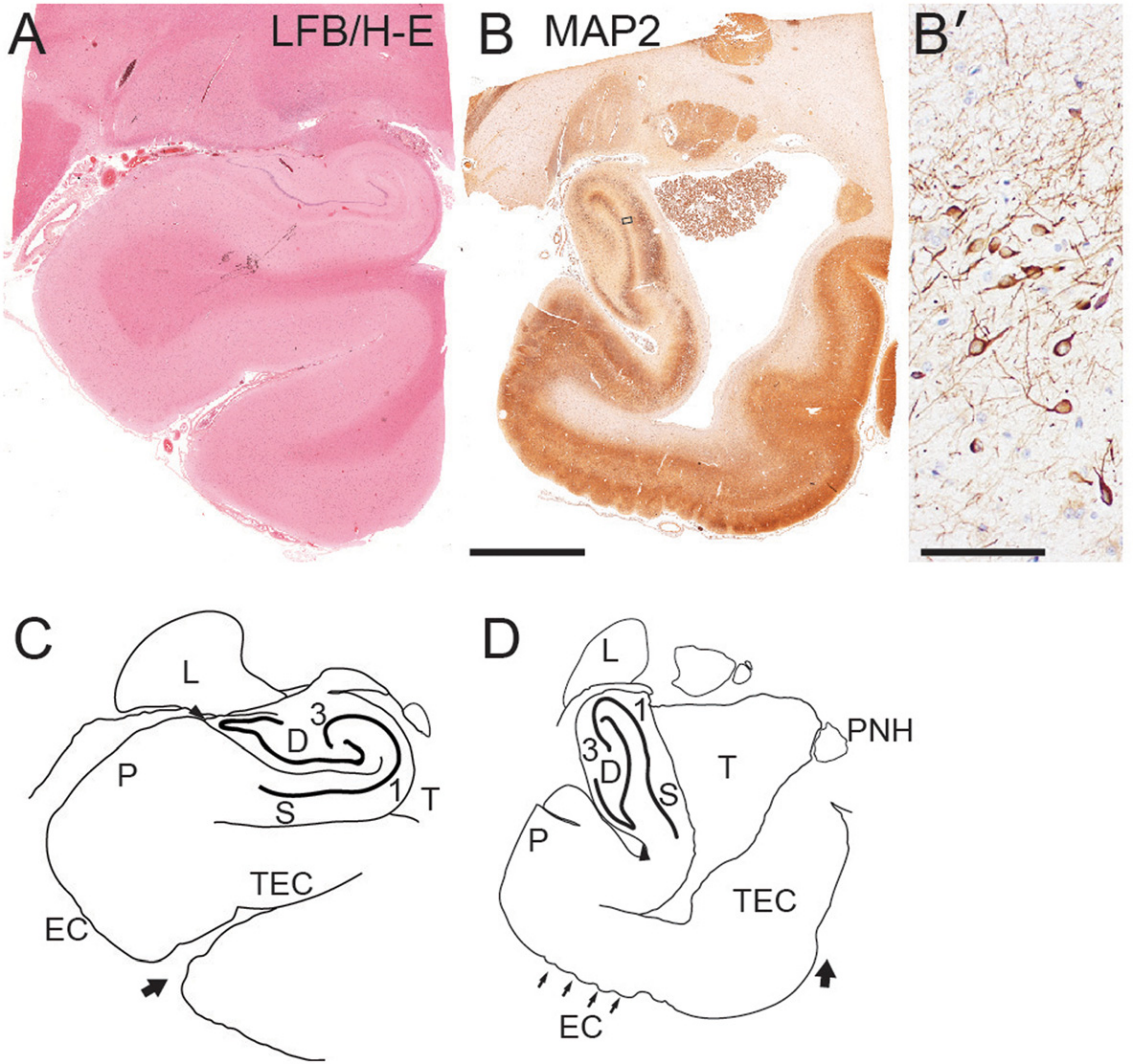
**Hippocampal malrotation. (A, C)** Normal control hippocampus (stained with Luxol fast blue, and hematoxylin and eosin) and interpretive line diagram. **(B, D)** Patient hippocampus (immunostained to detect MAP2) and diagram. **(B’)** Higher magnification of patient’s dentate gyrus showing reduced neuronal population. The patient’s hippocampus, including dentate gyrus **(D)**, CA1-CA3, and subiculum (S) was rotated by ~90 degrees from normal orientation. The temporal horn (T) of the lateral ventricle directly contacted the subiculum in patient but not control brain. The entorhinal cortex (EC), characterized by large neuron clusters in supragranular layers, appeared expanded in the patient brain (small arrows in **D**). Other abbreviations: P, presubiculum; L, lateral geniculate nucleus; PNH, periventricular nodular heterotopia; TEC, transentorhinal cortex. Large arrows indicate collateral sulcus. Scale bars: (in **B**) **A**-**D**, 750 μm; **B’**, 75 μm.

The bilateral hippocampi were malrotated through 90 degrees as viewed in a coronal plane (Figure 4). Major components of the archicortex (dentate gyrus, CA fields, and subiculum) were normally differentiated, but were oriented vertically rather than horizontally as in a normal control brain (Figure 4). The patient’s subiculum was not attached to subjacent white matter, but instead was in direct contact with the expanded lateral ventricle, suggesting persistence of the fetal ventricular anatomy (Figure 4B, D). This configuration contrasted with the normal age-matched control hippocampus, in which the subiculum contacts subjacent white matter contiguous to the entorhinal cortex (Figure 4A, C). The entire hippocampus was also atrophic and gliotic, with marked neuronal loss in all regions, including the dentate gyrus (Figure 4B’). Interestingly, the entorhinal cortex appeared possibly expanded, and the choroid plexus appeared relatively large (Figure 4B), although no systematic choroid plexus hyperplasia was noted on MRI or macroscopic brain examination.

### Chromosomal deletion mapping and affected genes

**Figure 5:**
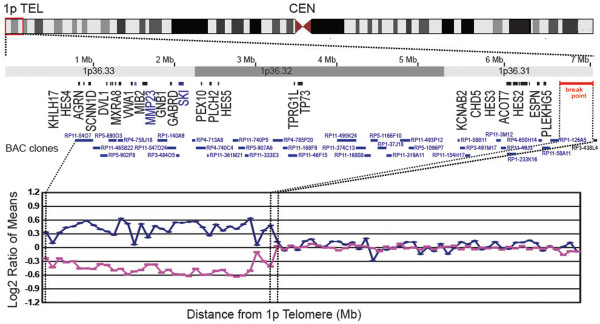
**Terminal deletion in the distal short arm of chromosome 1.** Microarray analysis showed a single-copy loss of 34 BAC clones from 1p36.3, spanning approximately 6.8 Mb, with break point between RP1-126A5 and RP3-438 L4. Probes are arranged with the most distal p-arm clones on the left. The blue tracing shows array comparative genomic hybridization data from the first microarray slide (reference Cy5/patient Cy3). The pink tracing shows data from the second microarray slide in which the dyes have been reversed (patient Cy5/reference Cy3). A subset of genes involved in neural development or function are indicated at their approximate position in the deleted region.

To gain further insights into the pathogenesis of brain and spinal malformations, we wished to map the chromosomal deletion with better resolution than provided by the clinical karyotype. Microarray analysis using 969 BAC clones covering 304 genetic loci on 41 chromosome arms disclosed single copy loss of DNA from 34 BAC clones, covering a contiguous segment of 1p36 from Rp11-54O7 to RP1-126A5 (Figure 5). This deletion included approximately 6.8 Mb of distal chromosome 1p, within the typical range of deletion sizes for this syndrome [1,3]. The deleted segment contains 112 known or predicted genes, including *CAMTA1*, which is located within the identified breakpoint region and may have been partially deleted (Figure 5, and Additional file 1) [25]. Many of the deleted genes have previously been implicated in nervous system development by studies in humans and mice (Table 1). For example, *Hes* genes are known to be critical for brain and spinal growth in mice [26,27], and four *HES* genes (*HES2*, *HES3*, *HES4*, *HES5*) were deleted in the present case. The potential roles of deleted genes in causing brain and spinal malformations in monosomy 1p36 will be discussed further below.

**Table 1:** Genes related to development and function of nervous system within region of 1p36.21-1p36.33 deletion

**Symbol**	**Description**	**Locus**	**Site**	**Mouse ortholog**	**Human diseases**	**Null mouse phenotype**	**Other**	**Refs.**
*KLHL17*	kelch-like 17 (Drosophila)	1p36.33	885,830..890,958	*Klhl17*			postsynaptic membrane	[28]
*HES4*	Hairy and enhancer of split 4 (Drosophila)	1p36.33	924,207..925,333*	**			*HES* gene family	[26,27]
*AGRN*	Agrin	1p36.33	945,366..981,355	*Agrn*	CMS (AR)	reduced NMJ		[29]
*SCNN1D*	sodium channel, nonvoltage-gated 1, delta	1p36.33	1,207,439..1,217,272	**			highly expressed in pyramidal neurons	[30]
*DVL1*	dishevelled, dsh homolog 1 (Drosophila)	1p36.33	1,260,521..1,274,358*	*Dvl1*	SJS (AR), CMT2A (AR)	abnormal social interactions		[31,32]
*MXRA8*	matrix-remodelling associated 8	1p36.33	1,277,934..1,283,778*	*Mxra8*			glia limitans/BBB	[33]
*VWA1*	von Willebrand factor A domain containing 1	1p36.33	1,360,772..1,366,009	*Vwa1*		peripheral nerve defects	ECM protein	[34]
*MIB2*	Mindbomb homolog 2 (Drosophila)	1p36.33	1,540,747..1,555,848	*Mib2*		exencephaly	ubiquitin ligase for Notch ligands	[35]
*MMP23B*	matrix metallopeptidase 23B	1p36.33	1,557,423..1,559,893	*Mmp23*	cranial suture closure***			[36]
*GNB1*	guanine nucleotide binding protein (G protein), beta polypeptide 1	1p36.33	1,716,725..1,822,526*	*Gnb1*		micrencephaly, NTDs	One of three beta subunits of G proteins	[37]
*GABRD*	GABA A receptor, delta	1p36.33	1,940,703..1,952,050	*Gabrd*	epilepsy	pro-epileptic		[38]
*SKI*	v-ski sarcoma viral oncogene homolog (avian)	1p36.33	2,149,994..2,229,316	*Ski*	facial clefting***	exencephaly, facial clefting, peripheral nerve myelination defects, PHPV, microphthalmos		[39,40]
*PEX10*	peroxisomal biogenesis factor 10	1p36.32	2,326,101..2,333,870*	*Pex10*	ALD (AR), ZS (AR)			[41]
*PLCH2*	phospholipase C, eta 2	1p36.32	2,397,614..2,426,824	*Plch2*	Mental retardation		neuron-specific isozyme	[42]
*HES5*	Hairy and enhancer of split 5 (Drosophila)	1p36.32	2,450,044..2,451,544*	*Hes5*		premature neuronal differentiation	synergistic with *Hes1*, *Hes3*	[26,27]
*TPRG1L*	tumor protein p63 regulated 1-like	1p36.32	3,531,416..3,536,555	*Tprg1l*			presynaptic protein	[43]
*TP73*	tumor protein p73	1p36.32	3,558,989..3,639,716	*Trp73*		Hippocampal, neocortical defects	apoptosis	[44,45]
*KCNAB2*	potassium voltage-gated channel, shaker-related subfamily, beta member 2	1p36.31	6,008,967..6,083,110	*Kcnab2*		Defects of learning and memory		[46]
*CHD5*	chromodomain helicase DNA binding protein 5	1p36.31	6,084,440..6,162,770*	*Chd5*			brain development, tumor suppressor gene	[47]
*HES3*	Hairy and enhancer of split 3 (Drosophila)	1p36.31	6,226,849..6,228,225	*Hes3*		premature differentiation	with *Hes1*, *Hes5*	[26,27]
*ACOT7*	acyl-CoA thioesterase 7	1p36.31	6,246,919..6,376,413*	*Acot7*			MTLE	[48]
*HES2*	Hairy and enhancer of split 2 (Drosophila)	1p36.31	6,397,883..6,402,566*	*Hes2*			expressed in developing nervous system	[26,27]
*ESPN*	Espin	1p36.31	6,407,435..6,443,591	*Espn*	congenital hearing loss (AR, AD)	jerker deafness	actin bundling	[49]
*PLEKG5*	pleckstrin homology domain containing, family G (with RhoGef domain) member 5	1p36.31	6,448,739..6,502,656*	*Plekhg*	lower motor neuron disease (AR)		activates NF kappa B	[50]

*AD* autosomal dominant, *ALD* adrenoleukodystrophy, *AR* autosomal recessive, *BBB* blood–brain barrier, *CMS* congenital myasthenic syndrome, *CMT2Ac* Charcot-Marie-Tooth disease type 2A, *ECM* extracellular matrix, *GABA* gamma-aminobutyric acid, *MTLE* mesial temporal lobe epilepsy, *NMJ* neuromuscular junction, *NTD* neural tube defect, *PHPV* persistent hyperplastic primary vitreous, *SJS* Schwartz-Jampel syndrome, *ZS* Zellweger syndrome. * complement = minus strand, ** none known, *** putative causative gene.

## Discussion

The current monosomy 1p36 case, with a 6.8 Mb subtelomeric deletion, presented with conspicuous neuropsychiatric abnormalities and other typical clinical manifestations of this syndrome [3,12]. The patient also had some disorders that are not typically associated with monosomy 1p36, namely transient conjugated hyperbilirubinemia in early infancy, and growth hormone deficiency. Postmortem neuropathological examination revealed significant brain and spinal malformations, including: (1) occipitotemporal PNHs; (2) bifrontal cortical dysgenesis resembling PMG; (3) callosal hypoplasia; (4) hippocampal malrotation; (5) small, anteriorly rotated vermis; (6) simplified inferior olives; and (7) hydromyelia in the lower thoracic and upper lumbar spinal cord. However, the hydromyelia was mild, and may have been secondary to cervicomedullary compression. None of the malformations were detected during life, and the last four have not previously been reported in monosomy 1p36. Thus, our findings suggest that brain and spinal malformations in monosomy 1p36 may have been underestimated in previous studies. The present study may help to raise awareness and increase detection of these malformations in monosomy 1p36 [51].

### Polymicrogyria-like cortical dysgenesis in monosomy 1p36

PMG is a poorly understood malformation characterized by excessive small gyri (often lending the cortex an irregular “Moroccan leather” surface appearance) and fusion of the molecular layer [52]. The first report of “possible polymicrogyria” in monosomy 1p36 was published in 1999 [14]. The presumed PMG in that case involved the left hemisphere, but no brain imaging or neuropathology was shown. More recently, it was reported that 13 of 64 (20%) monosomy 1p36 patients showed MRI evidence of PMG [10]. Interestingly, the apparent PMG affected perisylvian cortex preferentially, and involved the right hemisphere more often than the left. Also, the smallest deletion in that series was the last 4.8 Mb of 1p36, implying a critical region between BAC RP5-1096P7 and the 1p telomere. Subsequent studies have reported perisylvian PMG in three additional monosomy 1p36 patients [13,18,19]. Pachygyria and “coarse and nodular” cortex have also been described by neuroimaging in monosomy 1p36 [12]. However, none of the previous reports have documented the macroscopic brain pathology, or the histopathology of PMG in monosomy 1p36. Thus, the accuracy of the PMG diagnosis in monosomy 1p36, and its relation to other forms of PMG (not associated with monosomy 1p36), have remained uncertain.

Neuropathologists have distinguished several histological types of PMG on the basis of laminar morphology, but the significance of these variations is uncertain. The most common types of PMG have been described as “unlayered” and “four-layered”; less frequently, “two-layered” and “six-layered” PMG have been described [52-54]. In the present case, the cortical dysgenesis most resembled “six-layered” PMG. However, different PMG types may occur together in the same brain, and (despite their different morphologies) all seem to exhibit similar relative positions of layer-specific neurons [54]. Also, on neuroimaging, PMG may be difficult to distinguish from other MCDs, such as pachygyria and “cobblestone” malformations involving breaches in the pial surface of the brain, which can also occur together with PMG in the same brain [55]. Recently, it was proposed that fusion of the molecular layer is a defining feature of PMG [54]. By this criterion, cortical dysgenesis in the present case did not qualify as PMG, since little to no fusion of the molecular layer could be discerned (Figure 3). On the other hand, this is the only case of monosomy 1p36 with histopathological studies reported so far, and it is possible that authentic PMG with definite molecular layer fusion might occur in other cases; dysgenesis in the present case might represent a mild form of PMG. Alternatively, monosomy 1p36 may exhibit a unique form of “PMG-like” cortical dysgenesis, always lacking fusion of the molecular layer. Similarly to classic PMG [54], cortical dysgenesis in the present case showed no overall change in the relative positions of layer-specific neuron types (Figure 3H-K).

### Heterotopia in monosomy 1p36

Subcortical heterotopia are caused by defective cell migration from the fetal ventricular zone to the cortical plate, and can appear as scattered neurons, laminar bands, or PNH [52,56]. The first report of PNH in monosomy 1p36 appeared in 2006 [15]. In that case, multiple PNH were identified in the anterior portion of the left lateral ventricle. Subsequent reports have described multiple PNH bilaterally [9], and one PNH in the left lateral ventricle [18] of monosomy 1p36 patients. In the present case, multiple PNH were found in the temporal and occipital horns bilaterally (Figure. 1, 2 and 3). Thus, so far, there appears to be no consistent pattern in the distribution of PNH in monosomy 1p36, in contrast to some other forms of PNH, such as classic bilateral PNH linked to the *FLNA* gene on chromosome Xq28 [52].

Interestingly, laminar-like patterns are sometimes observed in PNH. In the present case, infragranular neurons (N52+ large pyramids) were located at the center of a nodule, and supragranular neurons (CR+) at the periphery (Figure 3M). Previous studies of PNH have reported disparate findings. One group reported that PNH contained predominantly supragranular neuron types, with very few infragranular-type neurons [57]. Another group found that PNH contained infragranular neurons at the periphery of nodules, with likely supragranular neurons occupying the center of nodules [58]. Altogether, the available data suggest that PNH may have variable composition, and may perhaps differ according to the genetic or other etiology of the malformation.

### Hippocampal malrotation in monosomy 1p36

Neuroimaging studies suggest that hippocampal malrotation occurs relatively frequently in association with other MCDs (22-56%), in seizure disorders (up to 43%), and possibly in otherwise healthy controls (0-10%) [59-64]. Furthermore, hippocampal malrotation is the most common abnormality of the hippocampus associated with febrile status epilepticus in children [64]. However, the overall significance of the aforementioned studies has been clouded by the use of different criteria to define hippocampal malrotation, and by a lack of correlations with histopathology to directly ascertain the direction and degree of malrotation, as well as any associated changes in the organization of hippocampal fields or subregions. In the present case, hippocampal malrotation (Figure 4) was associated with seizures. Furthermore, the morphology suggested an abnormal persistence of fetal configuration. During normal development, the ventricular surface of the subiculum comes into contact with the ventricular surface of the parahippocampal gyrus between 17–20 weeks of gestation [65], and these apposed surfaces fuse by the end of gestation [66] to form, in the mature brain, a solid core of white matter between the subiculum, entorhinal, and transentorhinal areas. Thus, the pathogenic mechanisms underlying hippocampal malrotation in this case may have included decreased hippocampal growth, increased intraventricular pressure, or defective fusion at the ventricular surfaces.

### Deleted genes and possible links to brain malformations

Like other subchromosomal deletion syndromes, monosomy 1p36 exhibits considerable phenotypic heterogeneity that may be attributed to multiple variables such as deletion size, genetic background, and intrauterine environment. Notably, the PMG-like cortical dysgenesis in monosomy 1p36 shows incomplete penetrance (~33%) [10] and variable distribution in the cerebral hemispheres and lobes [10,12-14,18,19]. Likewise, PNH seems to be variable in monosomy 1p36 [9,15,18]. Whereas the 1p36 region contains many genes that have been implicated in brain development or function (Table 1), some phenotypes presumably result from a combination of gene deficiencies. For these reasons, at present, it is impossible to definitively identify which gene deletions cause brain and spinal malformations in monosomy 1p36. However, reasonable candidate genes can be proposed on the basis of their previously defined functions in neurodevelopment, especially where critical regions have been defined. The brain phenotypes that seem most likely to have a genetic basis in the present case are micrencephaly, PMG-like cortical dysgenesis, and PNH. (As discussed above, hippocampal malrotation may have been secondary to ventriculomegaly and decreased overall brain growth).

Micrencephaly is a common finding in monosomy 1p36, affecting ~61% of patients [3], and was severe in the present case. Several important genes that regulate brain size, and neuronal and glial differentiation, were deleted in this patient: these included four *HES* genes (*HES2*, *HES3*, *HES4*, and *HES5*), and *GNB1*. *HES* (*hairy and enhancer of split*) genes are basic helix-loop-helix transcriptional repressors that mediate the highly conserved Notch signaling pathway, which, in the nervous system, controls cellular fate choices between neural stem cell (NSC) maintenance and differentiation of neurons and glia [26,27]. In mice, multiple *Hes* genes function in a redundant, gene dose-dependent manner to mediate canonical Notch signaling. Mice lacking multiple *Hes* genes or related effectors undergo accelerated embryonic neuronal differentiation causing a premature and transient excess of neurogenesis, followed by NSC depletion and premature cessation of neurogenesis [27,67]. *GNB1* (*guanine nucleotide binding protein [G protein], beta polypeptide 1*) encodes a G protein β subunit, one of five Gβ genes in the mouse and human genomes [37]. *Gnb1* deficient mice die prematurely in the embryonic or neonatal period with neural tube closure defects and micrencephaly [37]. The concurrent loss of one *GNB1* allele and four *HES* alleles has never been modeled in mice, but would presumably be likely to cause micrencephaly.

The penetrance of PMG-like cortical dysgenesis in monosomy 1p36 has been estimated at ~33%, and the phenotype appears to be linked to a putative critical region between 1.0 Mb and 4.8 Mb from the 1p telomere [10]. This critical region, which was entirely deleted in the present case, includes several genes that might contribute to the PMG-like phenotype. One such candidate is *TP73* (*tumor protein p73*), which encodes a member of the p53 family of transcription factors. In mice, *Trp73* (the *TP73* ortholog) is required for brain development. *Trp73*^−/−^ mice show congenital hydrocephalus, hippocampal dysgenesis, absence of the hippocampal sulcus (a folding defect), decreased expression of reelin and calretinin (markers of Cajal-Retzius neurons) in the cortical marginal zone and hippocampal molecular layer, frontal cortex hypoplasia, occipital cortex dyslamination, ventralized cortical patterning with expanded entorhinal-like cortex, increased cortical apoptosis, and probably aberrant connections [44,45]. Indeed, it was suggested [44] that “Extrapolation of the [*Trp73*^−/−^] mouse brain defect on the human brain would predict a substantial reduction of primary visual cortex and occipitotemporal association areas, a damage that, combined with the entorhinal malformation, would lead to severe cognitive disabilities and mental retardation.” Some defects in *Trp73*^−/−^ mice, such as the expanded entorhinal-like cortex, seemed to show parallels in the present case of monosomy 1p36 (Figures 3 and 4). However, PMG–like festooning of the supragranular layers was not observed in *Trp73*^−/−^ mice. Interestingly, human *TP73* is expressed monoallelically and is probably imprinted [68]. This fact raises the intriguing possibility that monosomy 1p36 may be associated with either complete loss or complete preservation of *TP73* expression, depending on whether the transcriptionally active parental allele was deleted. If *TP73* is imprinted and deficiency of *TP73* causes a brain malformation (e.g., entorhinal expansion, PMG-like dysgenesis, or PNH), then such phenotypes may be correlated with deletion of a specific parental allele in monosomy 1p36. On the other hand, *TP73* allele switching has been documented in some circumstances [68], and might provide a mechanism to counteract the effects of deleting an active *TP73* allele.

Another leading candidate gene in the putative critical region for PMG-like cortical dysgenesis is *PEX10* (*peroxisomal biogenesis factor 10*), which encodes a peroxisomal RING finger ubiquitin ligase required for peroxisome biogenesis [69]. Loss-of-function mutations in *PEX10* are one cause of Zellweger syndrome [69,70], a severe neurological disorder characterized by brain findings of centrosylvian PMG, medial pachygyria, subcortical heterotopias, and simplified inferior olivary nuclei [41]. However, Zellweger syndrome is a recessive disorder, and deletion of one *PEX10* allele would not account for brain malformations on its own. Nor was there any history of peroxisomal disorders in the patient’s family.

Reports of PNH in monosomy 1p36 are too few to estimate prevalence or define a critical region, but cases so far suggest limited expressivity with relatively few, small PNH. Usually categorized as a cell migration disorder, PNH may arise from disruptions of cytoskeletal proteins, vesicle trafficking, impaired cell adhesion, ependymal defects, or damage to radial glia [52,57,71]. Among genes deleted in the present case, *MXRA8* (*matrix-remodelling associated 8*) encodes an immunoglobulin superfamily cell adhesion molecule that, in adults, is localized to astrocyte endfeet that form the glia limitans and blood–brain barrier [33,72]. Although the developmental functions of *MXRA8* are unknown (since no gene deletion studies have been reported so far), one could speculate that it regulates cell migration or radial glia morphology. Another speculative candidate for contributing to PNH in monosomy 1p36 is *CHD5* (*chromodomain helicase DNA binding protein 5*), which encodes one component of a chromatin remodeling complex that is preferentially expressed in the nervous system [73]. In primary cultured rat neurons, shRNA-mediated knockdown of *Chd5* caused downregulation of some neuronal genes (such as *Fmr1*, *Dlx1*, and *Nefm*) and upregulation of others (such as *L1cam*, *Gabrd*, and *Ampd3*) [47]. Some of these changes, such as the upregulation of *L1cam* (which encodes an immunoglobulin superfamily cell adhesion molecule), might hypothetically interfere with cell migration and cause heterotopia. However, the developmental functions of *Chd5* have not been studied in knockout mice and currently remain unknown.

Many other genes deleted in the present case have been implicated in nervous system function and probably contribute to developmental delay, epilepsy, and related neuropsychiatric problems in monosomy 1p36 (Table 1; Additional file 1). However, genes that are expressed only in mature postmitotic neurons are less likely to be involved in the pathogenesis of malformations, and more likely to contribute to abnormalities of neuronal plasticity or function. Overall, in most cases of monosomy 1p36, neurological problems reflect perturbations of both developmental and mature brain functions.

## Conclusions

Our findings provide the first neuropathological evidence that multiple malformations of brain and spinal development occur in monosomy 1p36. The unique morphology of PMG-like cortical dysgenesis in the present case suggests that this lesion, in which fusion of the molecular layer was not evident (Figure 3), differs from classic PMG, in which fusion of the molecular layer is a defining feature [54]. More generally, our findings suggest that some types of PMG may exhibit unique morphologic features associated with different genetic and non-genetic etiologies. Finally, our analysis of the deleted chromosome region in this patient allowed us to not only identify candidate genes for malformations, but also propose a novel mechanism of phenotypic diversity in monosomy 1p36, namely the possibility that, due to imprinting, the penetrance of *TP73*-dependent phenotypes may be linked to deletion of a specific parental allele.

## Consent

Written informed consent was obtained from the patient’s parents for publication of this Case report and any accompanying images. A copy of the written consent is available for review by the Editor-in chief of this journal.

## Abbreviations

BAC: Bacterial artificial chromosome; CR: Calretinin; GFAP: Glial fibrillary acidic protein; MAP: Microtubule-associated protein; MRI: Magnetic resonance imaging; NSC: Neural stem cell; PDA: Patent ductus arteriosus; PMG: Polymicrogyria; PNH: Periventricular nodular heterotopia; VSD: Ventricular septal defect.

## Competing interests

There are no competing interests in the report.

## Authors’ contributions

NS, RAMD, WBD and RFH performed clinical and pathological studies and analysis. LGS carried out the genetic studies. AJB analyzed the MRI images. NS and RFH drafted the manuscript. All authors read and approved the final manuscript.

## Supplementary Material

Additional file 1 Genes within region of 1p36.21-1p36.33 deletion and references to gene functions. Click here for file
