# Supplementary material for: Neuropathology of brain and spinal malformations in a case of monosomy 1p36
Source: Acta Neuropathol Commun. 2013 Aug 2;1:45. doi: 10.1186/2051-5960-1-45 (PMC3893467; doi:10.1186/2051-5960-1-45)
Supplement: Additional file 1 — Genes within region of 1p36.21-1p36.33 deletion and references to gene functions. [file 2051-5960-1-45-S1.pdf]

Additional File 1 Genes within region of 1p36.21-1p36.33 deletion and references to gene functions

Supplementary to: N. Shiba et al., "Neuropathology of brain and spinal malformations in a case of monosomy 1p36," Acta Neuropathol Comm 2013. Correspondence: R. Hevner, Seattle Children's Research Inst., rhevner@uw.edu

Gene Symbol	Locus	Site	Type	Name	Mouse ortholog	Human Diseases	mouse phenotype	Other	Ref.
WASH5P	1p36.33	0000815..0019919*	pseudogene	WAS protein family homolog 5 pseudogene	**				
FAM138A	1p36.33	0023390..0025938*	miscRNA	family with sequence similarity 138, member A	**				
OR4F5	1p36.33	0058954..0059871*	protein coding	olfactory receptor, family 4, subfamily F, member 5	**				
OR4F16	1p36.33	0610959..0611897*	protein coding	olfactory receptor, family 4, subfamily F, member 16	**				
NCRNA00115	1p36.33	0751449..0752775*	miscRNA	non-protein coding RNA 115	**				
LOC643837	1p36.33	0766574..0782483	miscRNA	hypothetical LOC643837	**				
FAM41C	1p36.33	0793321..0795438*	miscRNA	family with sequence similarity 41, member C	**				
FLJ39609	1p36.33	0842818..0844680*	miscRNA	similar to hCG1995469	**				
SAMD11	1p36.33	0850984..0869824	protein coding	sterile alpha motif domain containing 11	Samd11				
NOC2L	1p36.33	0869446..0884542*	protein coding	nucleolar complex associated 2 homolog (S. cerevisiae)	Noc2l			HDAC-independent INHAT	
KLHL17	1p36.33	0885830..0890958	protein coding	kelch-like 17 (Drosophila)	Klhl17			postsynaptic membrane	[1]
PLEKHN1	1p36.33	0891740..0900351	protein coding	pleckstrin homology domain containing, family N member 1	Plekhn1				
HES4	1p36.33	0924207..0925333*	protein coding	hairy and enhancer of split 4 (Drosophila)	**			HES gene family	[2, 3]
ISG15 (G1P2)	1p36.33	0938742..0939782	protein coding	ISG15 ubiquitin-like modifier	Isg15(G1p2)		vialble and fertile	immune response to Mycobacteria	[4]
AGRN	1p36.33	0945366..0981355	protein coding	agrin	Agrn	CMS (AR)	reduced NMJ		[5, 6]
C1orf159	1p36.33	1007068..1041332*	protein coding	chromosome 1 open reading frame 159	**				
TTLL10	1p36.33	1104940..1111104	protein coding	tubulin tyrosine ligase-like family, member 10	Ttll10			glycine ligase	
TNFRSF18 (GITR)	1p36.33	1128751..1131952*	protein coding	tumor necrosis factor receptor superfamily, member 18	Tnfrsf18		immune defects	immunological self-tolerance	[7, 8]
TNFRSF4 (OX40)	1p36.33	1136569..1139375*	protein coding	tumor necrosis factor receptor superfamily, member 4	Tnfrsf4		immune defects	suppresses apoptosis	[9, 10]
SDF4	1p36.33	1142151..1157274*	protein coding	stromal cell derived factor 4	Sdf4				
B3GALT6	1p36.33	1157492..1160284	protein coding	UDP-Gal:betaGal beta 1,3-galactosyltransferase polypeptide (	B3galt6			GAG Biosynthesis	[11]
FAM132A	1p36.33	1167696..1171965*	protein coding	family with sequence similarity 132, member A	Fam132a				
UBE2J2	1p36.33	1179155..1199097*	protein coding	ubiquitin-conjugating enzyme E2, J2 (UBC6 homolog, yeast)	Ube2j2				
LOC100128842	1p36.33	1184557..1186958*	miscRNA	hypothetical protein LOC100128842	**				
SCNN1D (ENaCd)	1p36.33	1207439..1217272	protein coding	sodium channel, nonvoltage-gated 1, delta	**			highly expressed in pyramidal neurons	[12, 13]
ACAP3	1p36.33	1218807..1228503*	protein coding	ArfGAP with coiled-coil, ankyrin repeat and PH domains 3	Acap3				
PUSL1	1p36.33	1233857..1236920	protein coding	pseudouridylate synthase-like 1	Pusl1				
CPSF3L	1p36.33	1236828..1249909*	protein coding	cleavage and polyadenylation specific factor 3-like	Cpsf3l			subunit of Integrator snRNA processing	[14]
GLTPD1	1p36.33	1250006..1254139	protein coding	glycolipid transfer protein domain containing 1	Gltpd1				
TAS1R3	1p36.33	1256589..1259707	protein coding	taste receptor, type 1, member 3	Tas1r3		taste perception defect	GPCR; calcium detection by taste	[15]
DVL1	1p36.33	1260521..1274358*	protein coding	dishevelled, dsh homolog 1 (Drosophila)	Dvl1	SJS (AR), CMT2A (AR)	abnormal social interactions,	abnormal sensorimotor gating	[16, 17]
MXRA8	1p36.33	1277934..1283778*	protein coding	matrix-remodelling associated 8	Mxra8			glia limitans / BBB	[18]
AURKAIP1	1p36.33	1298973..1300443*	protein coding	aurora kinase A interacting protein 1	Aurkaip1				
CCNL2	1p36.33	1310954..1324553*	protein coding	cyclin L2	Ccnl2				
LOC148413	1p36.33	1324795..1328199	miscRNA	hypothetical LOC148413	**				
MRPL20	1p36.33	1327159..1332524*	protein coding	mitochondrial ribosomal protein L20	Mrpl20				
hCG_20426	1p36.33	1343665..1346513*	protein coding	hypothetical protein LOC441869	**				
TMEM88B	1p36.33	1351425..1353030	protein coding	Transmembrane protein 88B	A230069A22Rik				
VWA1	1p36.33	1360772..1366009	protein coding	von Willebrand factor A domain containing 1	Vwa1		peripheral nerve defects	ECM protein	[19]
ATAD3C	1p36.33	1387910..1395401	protein coding	ATPase family, AAA domain containing 3C	**				
ATAD3B	1p36.33	1397027..1421445	protein coding	ATPase family, AAA domain containing 3B	**			Mitochondrial nucleoid organization	[20, 21]
ATAD3A	1p36.33	1437418..1459927	protein coding	ATPase family, AAA domain containing 3A	Atad3a			Mitochondrial nucleoid organization	[20, 21]
C1orf70	1p36.33	1460602..1465597*	protein coding	chromosome 1 open reading frame 70	EG381582				
SSU72	1p36.33	1466916..1500125*	protein coding	SSU72 RNA polymerase II CTD phosphatase homolog (S. cer	Ssu72				
MIB2	1p36.33	1540747..1555848	protein coding	mindbomb homolog 2 (Drosophila)	Mib2		exencephaly	ubiquitin ligase for Notch ligands	[22]
MMP23B	1p36.33	1557423..1559893	protein coding	matrix metallopeptidase 23B	Mmp23	cranial suture closure***			[23]
CDK11A (CDC2L2)	1p36.33	1560463..1580350*	protein coding	cell division cycle 2-like 2 (PITSLRE proteins)	Cdk11b			sister chromatid cohesion; apoptosis	[24-26]
RP11-345P4.4	1p36.33	1582802..1614103*	protein coding	similar to solute carrier family 35, member E2	A530082C11Rik				
MMP23A	1p36.33	1619121..1623110	pseudogene	matrix metallopeptidase 23A (pseudogene)	**				
CDK11B (CDC2L1)	1p36.33	1624180..1645637*	protein coding	cell division cycle 2-like 1 (PITSLRE proteins)	Cdk11b			centriole duplication	[24-26]
SLC35E2	1p36.33	1653541..1667291*	protein coding	solute carrier family 35, member E2	**				
NADK	1p36.33	1672531..1699769*	protein coding	NAD kinase	Nadk				
GNB1	1p36.33	1706589..1812355*	protein coding	guanine nucleotide binding protein (G protein), beta polypeptic	Gnb1		micrencephaly, NTDs	One of three beta subunits of G proteins	[27]
CALML6 (CAGLP)	1p36.33	1836126..1838593	protein coding	calmodulin-like 6	**				
TMEM52	1p36.33	1838890..1840567*	protein coding	transmembrane protein 52	Tmem52				
C1orf222	1p36.33	1843256..1849228*	protein coding	chromosome 1 open reading frame 222	**				
KIAA1751	1p36.33	1874612..1925136*	protein coding	KIAA1751	2010015L04Rik				
GABRD	1p36.33	1940703..1952050	protein coding	gamma-aminobutyric acid (GABA) A receptor, delta	Gabrd	epilepsy	pro-epileptic		[28 ,29]
PRKCZ	1p36.33	1971769..2106694	protein coding	protein kinase C, zeta	Prkcz		lymphoid defects	aPKC (Ca++ independent)	[30]
C1orf86	1p36.33	2105777..2116074*	protein coding	chromosome 1 open reading frame 86	2610002J02Rik				
LOC100128003	1p36.33	2110342..2114884	miscRNA	hypothetical protein LOC100128003	**				
SKI	1p36.33	2149994..2229316	protein coding	v-ski sarcoma viral oncogene homolog (avian)	Ski	facial clefting***	exencephaly, facial clefting,	peripheral nerve myelination deficiency, PHPV	[31, 32]
MORN1	1p36.33-p36.32	2242556..2312853*	protein coding	MORN repeat containing 1	Morn1				
LOC100129534	1p36.33	2271716..2273960*	pseudo	small nuclear ribonucleoprotein polypeptide N pseudogene	**				
RER1	1p36.32	2313074..2326734	protein coding	RER1 retention in endoplasmic reticulum 1 homolog (S. cerev	Rer1				
PEX10	1p36.32	2326101..2333870*	protein coding	peroxisomal biogenesis factor 10	Pex10	adrenoleukodystrophy (AR), Zellweger syndrome (AR)			[33, 34]
PLCH2	1p36.32	2397614..2426824	protein coding	phospholipase C, eta 2	Plch2	mental retardation		neuron-specific isozyme	[35, 36]
PANK4	1p36.32	2429835..2447895*	protein coding	pantothenate kinase 4	Pank4				
HES5	1p36.32	2450044..2451544*	protein coding	hairy and enhancer of split 5 (Drosophila)	Hes5		premature differentiation	HES gene family	[2, 3]
LOC115110	1p36.32	2471219..2474144*	miscRNA	hypothetical protein LOC115110	**				
TNFRSF14 (HVEM)	1p36.32	2479150..2486613*	protein coding	tumor necrosis factor receptor superfamily, member 14 (herpe	Tnfrsf14		immune defects	T cell regulation	[37, 38]
C1orf93	1p36.32	2508109..2512762	protein coding	chromosome 1 open reading frame 93	2810405K02Rik				
MMEL1 (NEP2)	1p36.32	2511941..2554289*	protein coding	membrane metallo-endopeptidase-like 1	Mmel1				
ACTRT2	1p36.32	2927906..2929327	protein coding	actin-related protein T2	Actrt2				

FLJ42875	1p36.32	2966041..2974187*	miscRNA	hypothetical LOC440556	**				
PRDM16	1p36.32	2975604..3345045	protein coding	PR domain containing 16	Prdm16		heterochromatin disintegratio	Zinc finger transcription factor	[39]
ARHGEF16	1p36.32	3369982..3387537	protein coding	Rho guanine exchange factor (GEF) 16	Arhgef16				
MEGF6	1p36.32	3394366..3517919*	protein coding	multiple EGF-like-domains 6	Megf6				
TPRG1L	1p36.32	3531416..3536555	protein coding	tumor protein p63 regulated 1-like	Tprgl			presynaptic protein	[40]
WDR8	1p36.32	3537199..3556497*	protein coding	WD repeat domain 8	Wdr8				
TP73	1p36.32	3558989..3639716	protein coding	tumor protein p73	Trp73		hippocampal dysgenesis, cor	apoptosis	[41, 42]
KIAA0495	1p36.32	3642408..3653746*	protein coding	KIAA0495	**				
CCDC27	1p36.32	3658822..3678069	protein coding	coiled-coil domain containing 27	Ccdc27				
LOC388588	1p36.32	3679212..3682379	miscRNA	hypothetical LOC388588	**				
LRRC47	1p36.32	3686644..3702928*	protein coding	leucine rich repeat containing 47	Lrrc47				
KIAA0562	1p36.32	3721204..3763657*	protein coding	KIAA0562	BC046331				
DFFB	1p36.32	3763705..3791853	protein coding	DNA fragmentation factor, 40kDa, beta polypeptide (caspase-a	Dffb		impaired thymic development	apoptotic DNA degradation	[43]
C1orf174	1p36.32	3795557..3806709*	protein coding	chromosome 1 open reading frame 174	A430005L14Rik				
LOC100133612	1p36.32	3806828..3821869	miscRNA	similar to hCG1815312, non-coding RNA	**				
LOC284661	1p36.32	4371971..4384604	miscRNA	hypothetical LOC284661	**				
AJAP1 (SHREW1)	1p36.32	4614965..4743711	protein coding	adherens junctions associated protein 1	Ajap1				
NPHP4	1p36.31	5845457..5975118*	protein coding	nephronophthisis 4	Nphp4	juvenile nephronophthisis (AR	retinal degeneration	primary cilium	[44, 45]
KCNAB2	1p36.31	6008967..6083110	protein coding	potassium voltage-gated channel, shaker-related subfamily, be	Kcnab2		memory impairment		[46]
CHD5	1p36.31	6084440..6162770*	protein coding	chromodomain helicase DNA binding protein 5	Chd5			brain development, tumor suppressor gene,	[47]
RPL22	1p36.31	6167667..6182266*	protein coding	ribosomal protein L22	Rpl22				
RNF207	1p36.31	6188776..6203946	protein coding	ring finger protein 207	Rnf207				
ICMT	1p36.21	6203840..6218631*	protein coding	isoprenylcysteine carboxyl methyltransferase	Icmt		embryonic lethal	targets proteins to cell membrane	[48, 49]
HES3	1p36.31	6226849..6228225	protein coding	hairy and enhancer of split 3 (Drosophila)	Hes3		premature differentiation	HES gene family	[2, 3]
GPR153	1p36.31	6231443..6243622*	protein coding	G protein-coupled receptor 153	Gpr153				
ACOT7 (BACH)	1p36.31	6246919..6376413*	protein coding	acyl-CoA thioesterase 7	Acot7			MTLE	[50]
HES2	1p36.31	6397883..6402566*	protein coding	hairy and enhancer of split 2 (Drosophila)	Hes2			expressed in developing nervous system	[2, 3]
ESPN	1p36.31	6407435..6443591	protein coding	espin	Espn (je)	congenital hearing loss (AD, A	jerker deafness	actin bundling	[51, 52]
TNFRSF25 (DR3)	1p36.31	6443798..6448842*	protein coding	tumor necrosis factor receptor superfamily, member 25	Tnfrsf25	rheumatoid arthritis	immune defects	T cell apoptosis and self-tolerance	[53-55]
PLEKHG5	1p36.31	6448739..6502656*	protein coding	pleckstrin homology domain containing, family G (with RhoGe	Plekhg5	lower motor neuron disease (AR)		activates NF kappa B	[56]
NOL9	1p36.31	6507792..6537168*	protein coding	nucleolar protein 9	Nol9				
TAS1R1	1p36.31	6538021..6562404	protein coding	taste receptor, type 1, member 1	Tas1r1		taste perception defect	GPCR; sweet and umami tastes	[57]
ZBTB48 (HKR3)	1p36.31	6562698..6571926	protein coding	zinc finger and BTB domain containing 48	Zbtb48				
KLHL21	1p36.31	6573371..6585516*	protein coding	kelch-like 21 (Drosophila)	Klhl21				
PHF13	1p36.31	6596332..6606643	protein coding	PHD finger protein 13	Phf13				
THAP3	1p36.31	6607839..6618232	protein coding	THAP domain containing, apoptosis associated protein 3	Thap3				
DNAJC11	1p36.31	6616818..6684460*	protein coding	DnaJ (Hsp40) homolog, subfamily C, member 11	Dnajc11				
CAMTA1	1p36.31-p36.23	6767971..7752351	protein coding	calmodulin-binding transcription activator 1	Camta1				

AD autosomal dominant, AR autosomal recessive, BBB blood brain barrier, CMS Congenital myasthenic syndrome, CMT2A Charcot-Marie-Tooth disease type 2A, ECM extracellular matrix protein, GABA Gamma-aminobutyric acid, GPCR G protein-coupled receptor, HDAC hyston deacetylases, IHAT inhibitor of histone acetyltransferase, MTLE mesial temporal epilepsy, PHPV persistent hyperplastic primary vitreous, SJS Schwartz-Jampel syndrome, SLS Senior-Loken syndrome
* complement=minus strand, ** not exist, *** putative causative gene

References for Additional File 1 Table

1. Chen Y, Derin R, Petralia RS, Li M: **Actinfilin, a brain-specific actin-binding protein in postsynaptic density**. *J Biol Chem* 2002, **277**:30495-30501.
2. Kageyama R, Ohtsuka T, Kobayashi T: **The *Hes* gene family: repressors and oscillators that orchestrate embryogenesis**. *Development* 2007, **134**:1243-1251.
3. Kageyama R, Ohtsuka T, Kobayashi T: **Roles of *Hes* genes in neural development**. *Dev Growth Differ* 2008, **50**(Suppl 1):S97-S103.
4. Bogunovic D, Byun M, Durfee LA, Abhyankar A, Sanal O, Mansouri D, Salem S, Radovanovic I, Grant AV, Adimi P, Mansouri N, Okada S, Bryant VL, Kong XF, Kreins A, Velez MM, Boisson B, Khalilzadeh S, Ozcelik U, Darazam IA, Schoggins JW, Rice CM, Al-Muhsen S, Behr M, Vogt G, Puel A, Bustamante J, Gros P, Huibregtse JM, Abel L, Boisson-Dupuis S, Casanova JL: **Mycobacterial disease and impaired IFN-γ immunity in humans with inherited ISG15 deficiency**. *Science* 2012, **337**:1684-1688.
5. Gautam M, Noakes PG, Moscoso L, Rupp F, Scheller RH, Merlie JP, Sanes JR: **Defective neuromuscular synaptogenesis in agrin-deficient mutant mice**. *Cell* 1996, **85**:525-535.
6. Huzé C, Bauché S, Richard P, Chevessier F, Goillot E, Gaudon K, Ben Ammar A, Chaboud A, Grosjean I, Lecuyer HA, Bernard V, Rouche A, Alexandri N, Kuntzer T, Fardeau M, Fournier E, Brancaccio A, Rüegg MA, Koenig J, Eymard B, Schaeffer L, Hantaï D: **Identification of an agrin mutation that causes congenital myasthenia and affects synapse function**. *Am J Hum Genet* 2009, **85**:155-167.
7. Ronchetti S, Nocentini G, Riccardi C, Pandolfi PP: **Role of GITR in activation response of T lymphocytes**. *Blood* 2002, **100**:350-352.
8. Shimizu J, Yamazaki S, Takahashi T, Ishida Y, Sakaguchi S: **Stimulation of CD25(+)CD4(+) regulatory T cells through GITR breaks immunological self-tolerance**. *Nat Immunol* 2002, **3**:135-142.
9. Kopf M, Ruedl C, Schmitz N, Gallimore A, Lefrang K, Ecabert B, Odermatt B, Bachmann MF: **OX40-deficient mice are defective in Th cell proliferation but are competent in generating B cell and CTL responses after virus infection**. *Immunity* 1999, **11**:699-708.
10. Pippig SD, Peña-Rossi C, Long J, Godfrey WR, Fowell DJ, Reiner SL, Birkeland ML, Locksley RM, Barclay AN, Killeen N: **Robust B cell immunity but impaired T cell proliferation in the absence of CD134 (OX40).** *J Immunol* 1999, **163**:6520-6529.

11. Bai X, Zhou D, Brown JR, Crawford BE, Hennet T, Esko JD: **Biosynthesis of the linkage region of glycosaminoglycans: cloning and activity of galactosyltransferase II, the sixth member of the beta 1,3-galactosyltransferase family (beta 3GalT6).** *J Biol Chem* 2001, **276**: 48189-48195.
12. Giraldez T, Afonso-Oramas D, Cruz-Muros I, Garcia-Marin V, Pagel P, González-Hernández T, Alvarez de la Rosa D: **Cloning and functional expression of a new epithelial sodium channel delta subunit isoform differentially expressed in neurons of the human and monkey telencephalon**. *J Neurochem* 2007, **102**:1304-1315.
13. Yamamura H, Ugawa S, Ueda T, Nagao M, Shimada S: **Protons activate the delta-subunit of the epithelial Na+ channel in humans**. *J Biol Chem* 2004, **279**:12529-12534.
14. Baillat D, Hakimi MA, Naar AM, Shilatifard A, Cooch N, Shiekhattar R: **Integrator, a multiprotein mediator of small nuclear RNA processing, associates with the C-terminal repeat of RNA polymerase II**. *Cell* 2005, **123**:265-276.
15. Tordoff MG, Alarcón LK, Valmeki S, Jiang P: **T1R3: a human calcium taste receptor**. *Sci Rep* 2012, **2**:496.
16. Ahmad-Annuar A, Ciani L, Simeonidis I, Herreros J, Fredj NB, Rosso SB, Hall A, Brickley S, Salinas PC: **Signaling across the synapse: a role for Wnt and Dishevelled in presynaptic assembly and neurotransmitter release**. *J Cell Biol* 2006, **174**:127-139.
17. Long JM, LaPorte P, Paylor R, Wynshaw-Boris A: **Expanded characterization of the social interaction abnormalities in mice lacking *Dvl1***. *Genes Brain Behav* 2004, **3**:51-62.
18. Yonezawa T, Ohtsuka A, Yoshitaka T, Hirano S, Nomoto H, Yamamoto K, Ninomiya Y: **Limitrin, a novel immunoglobulin superfamily protein localized to glia limitans formed by astrocyte endfeet**. *Glia* 2003, **44**:190-204.
19. Allen JM, Zamurs L, Brachvogel B, Schlötzer-Schrehardt U, Hansen U, Lamandé SR, Rowley L, Fitzgerald J, Bateman JF: **Mice lacking the extracellular matrix protein WARP develop normally but have compromised peripheral nerve structure and function**. *J Biol Chem* 2009, **284**:12020-12030.
20. He J, Mao CC, Reyes A, Sembongi H, Di Re M, Granycome C, Clippingdale AB, Fearnley IM, Harbour M, Robinson AJ, Reichelt S, Spelbrink JN, Walker JE, Holt IJ: **The AAA+ protein ATAD3 has displacement loop binding properties and is involved in mitochondrial nucleoid organization**. *J Cell Biol* 2007, **176**:141-146.
21. Merle N, Féraud O, Gilquin B, Hubstenberger A, Kieffer-Jacquinot S, Assard N, Bennaceur-Griscelli A, Honnorat J, Baudier J: **ATAD3B is a human embryonic stem cell**

specific mitochondrial protein, re-expressed in cancer cells, that functions as dominant negative for the ubiquitous ATAD3A. *Mitochondrion* 2012, **12**:441-8.

22. Wu JI, Rajendra R, Barsi JC, Durfee L, Benito E, Gao G, Kuruvilla M, Hrdlicková R, Liss AS, Artzt K: **Targeted disruption of Mib2 causes exencephaly with a variable penetrance**. *Genesis* 2007, **45**:722-727.
23. Gajecka M, Yu W, Ballif BC, Glotzbach CD, Bailey KA, Shaw CA, Kashork CD, Heilstedt HA, Ansel DA, Theisen A, Rice R, Rice DP, Shaffer LG: **Delineation of mechanisms and regions of dosage imbalance in complex rearrangements of 1p36 leads to a putative gene for regulation of cranial suture closure**. *Eur J Hum Genet* 2005, **13**:139-149.
24. Franck N, Montembault E, Romé P, Pascal A, Cremet JY, Giet R: **CDK11(p58) is required for centriole duplication and Plk4 recruitment to mitotic centrosomes**. *PLoS One* 2011, **6**:e14600.
25. Hu D, Valentine M, Kidd VJ, Lahti JM: **CDK11(p58) is required for the maintenance of sister chromatid cohesion**. *J Cell Sci* 2007, **120**:2424-2434.
26. Shi J, Hershey JW, Nelson MA: **Phosphorylation of the eukaryotic initiation factor 3f by cyclin-dependent kinase 11 during apoptosis**. *FEBS Lett* 2009, **583**:91-97.
27. Okae H, Iwakura Y: **Neural tube defects and impaired neural progenitor cell proliferation in gbeta1-deficient mice**. *Dev Dyn* 2010, **239**:1089-1101
28. Dibbens LM, Feng HJ, Richards MC, Harkin LA, Hodgson BL, Scott D, Jenkins M, Petrou S, Sutherland GR, Scheffer IE, Berkovic SF, Macdonald RL, Mulley JC: ***GABRD* encoding a protein for extra- or peri-synaptic GABAA receptors is a susceptibility locus for generalized epilepsies**. *Hum Mol Genet* 2004, **13**:1315-1319.
29. Windpassinger C, Kroisel PM, Wagner K, Petek E: **The human gamma-aminobutyric acid A receptor delta (GABRD) gene: molecular characterisation and tissue-specific expression**. *Gene* 2002, **292**:25-31.
30. Martin P, Duran A, Minguet S, Gaspar ML, Diaz-Meco MT, Rennert P, Leitges M, Moscat J: **Role of zeta PKC in B-cell signaling and function**. *EMBO J* 2002, **21**:4049-4057.
31. Atanasoski S, Notterpek L, Lee HY, Castagner F, Young P, Ehrengruber MU, Meijer D, Sommer L, Stavnezer E, Colmenares C, Suter U: **The protooncogene *Ski* controls Schwann cell proliferation and myelination**. *Neuron* 2004, **43**:499-511.
32. Colmenares C, Heilstedt HA, Shaffer LG, Schwartz S, Berk M, Murray JC, Stavnezer E: **Loss of the *SKI* proto-oncogene in individuals affected with 1p36 deletion syndrome is predicted by strain-dependent defects in *Ski*-/- mice**. *Nat Genet* 2002, **30**:106-109.

33. Suzuki Y, Shimozawa N, Imamura A, Fukuda S, Zhang Z, Orii T, Kondo N: **Clinical, biochemical and genetic aspects and neuronal migration in peroxisome biogenesis disorders**. *J Inherit Metab Dis* 2001, **24**:151-165.
34. Warren DS, Wolfe BD, Gould SJ: **Phenotype-genotype relationships in PEX10-deficient peroxisome biogenesis disorder patients**. *Hum Mutat* 2000, **15**:509-521.
35. Lo Vasco VR: **Role of phosphoinositide-specific phospholipase C η2 in isolated and syndromic mental retardation**. *Eur Neurol* 2011, **65**:264-269.
36. Nakahara M, Shimozawa M, Nakamura Y, Irino Y, Morita M, Kudo Y, Fukami K: **A novel phospholipase C, PLC(eta)2, is a neuron-specific isozyme**. *J Biol Chem* 2005, **280**:29128-29134.
37. Chen L, Flies DB: **Molecular mechanisms of T cell co-stimulation and co-inhibition**. *Nat Rev Immunol* 2013, **13**:227-242.
38. Wang Y, Subudhi SK, Anders RA, Lo J, Sun Y, Blink S, Wang Y, Wang J, Liu X, Mink K, Degrandi D, Pfeffer K, Fu YX: **The role of herpesvirus entry mediator as a negative regulator of T cell-mediated responses**. *J Clin Invest* 2005, **115**:711-717.
39. Pinheiro I, Margueron R, Shukeir N, Eisold M, Fritzsch C, Richter FM, Mittler G, Genoud C, Goyama S, Kurokawa M, Son J, Reinberg D, Lachner M, Jenuwein T: **Prdm3 and Prdm16 are H3K9me1 methyltransferases required for mammalian heterochromatin integrity**. *Cell* 2012, **150**:948-960.
40. Kremer T, Kempf C, Wittenmayer N, Nawrotzki R, Kuner T, Kirsch J, Dresbach T: **Mover is a novel vertebrate-specific presynaptic protein with differential distribution at subsets of CNS synapses**. *FEBS Lett* 2007, **581**:4727-4733.
41. Meyer G, Cabrera Socorro A, Perez Garcia C G, Martinez Millan L, Walker N, Caput D: **Developmental roles of p73 in Cajal-Retzius cells and cortical patterning**. *J Neurosci* 2004, **24**:9878-9887.
42. Yang A, Walker N, Bronson R, Kaghad M, Oosterwegel M, Bonnin J, Vagner C, Bonnet H, Dikkes P, Sharpe A, McKeon F, Caput D: **p73-deficient mice have neurological, pheromonal and inflammatory defects but lack spontaneous tumours**. *Nature* 2000, **404**:99-103.
43. Kawane K, Fukuyama H, Yoshida H, Nagase H, Ohsawa Y, Uchiyama Y, Okada K, Iida T, Nagata S: **Impaired thymic development in mouse embryos deficient in apoptotic DNA degradation**. *Nat Immunol* 2003, **4**:138-144.
44. Schuermann MJ, Otto E, Becker A, Saar K, Rüschendorf F, Polak BC, Ala-Mello S, Hoefele J, Wiedensohler A, Haller M, Omran H, Nürnberg P, Hildebrandt F: **Mapping of**

gene loci for nephronophthisis type 4 and Senior-Løken syndrome, to chromosome 1p36. *Am J Hum Genet* 2002, **70**:1240-1246.

45. Won J, Marín de Evsikova C, Smith RS, Hicks WL, Edwards MM, Longo-Guess C, Li T, Naggert JK, Nishina PM: ***NPHP4* is necessary for normal photoreceptor ribbon synapse maintenance and outer segment formation, and for sperm development**. *Hum Mol Genet* 2011, **20**:482-496.
46. Perkowski JJ, Murphy GG: **Deletion of the mouse homolog of KCNAB2, a gene linked to monosomy 1p36, results in associative memory impairments and amygdala hyperexcitability**. *J Neurosci* 2011, **31**:46-54.
47. Thompson PM, Gotoh T, Kok M, White PS, Brodeur GM: ***CHD5*, a new member of the chromodomain gene family, is preferentially expressed in the nervous system**. *Oncogene* 2003, **22**:1002-1011.
48. Bergo MO, Leung GK, Ambroziak P, Otto JC, Casey PJ, Gomes AQ, Seabra MC, Young SG: **Isoprenylcysteine carboxyl methyltransferase deficiency in mice**. *J Biol Chem* 2001, **276**:5841-5845.
49. Dai Q, Choy E, Chiu V, Romano J, Slivka SR, Steitz SA, Michaelis S, Philips MR: **Mammalian prenylcysteine carboxyl methyltransferase is in the endoplasmic reticulum**. *J Biol Chem* 1998, **273**:15030-15034.
50. Yang JW, Czech T, Yamada J, Csaszar E, Baumgartner C, Slavc I, Lubec G: **Aberrant cytosolic acyl-CoA thioester hydrolase in hippocampus of patients with mesial temporal lobe epilepsy**. *Amino Acids* 2004, **27**:269-275.
51. Donaudy F, Zheng L, Ficarella R, Ballana E, Carella M, Melchionda S, Estivill X, Bartles JR, Gasparini P: **Espin gene (*ESPN*) mutations associated with autosomal dominant hearing loss cause defects in microvillar elongation or organisation**. *J Med Genet* 2006, **43**:157-161.
52. Naz S, Griffith AJ, Riazuddin S, Hampton LL, Battey JF Jr, Khan SN, Riazuddin S, Wilcox ER, Friedman TB: **Mutations of ESPN cause autosomal recessive deafness and vestibular dysfunction**. *J Med Genet* 2004, **41**:591-595.
53. Bayry J: **Immunology: TL1A in the inflammatory network in autoimmune diseases**. *Nat Rev Rheumatol* 2010, **6**:67-68.
54. Bull MJ, Williams AS, Mecklenburgh Z, Calder CJ, Twohig JP, Elford C, Evans BA, Rowley TF, Slebioda TJ, Taraban VY, Al-Shamkhani A, Wang EC: **The Death Receptor 3-TNF-like protein 1A pathway drives adverse bone pathology in inflammatory arthritis**. *J Exp Med* 2008, **205**:2457-2464.

55. Wang EC, Thern A, Denzel A, Kitson J, Farrow SN, Owen MJ: **DR3 regulates negative selection during thymocyte development**. *Mol Cell Biol* 2001, **21**:3451-3461.
56. Maystadt I, Rezsöhazy R, Barkats M, Duque S, Vannuffel P, Remacle S, Lambert B, Najimi M, Sokal E, Munnich A, Viollet L, Verellen-Dumoulin C: **The nuclear factor kappaB-activator gene PLEKHG5 is mutated in a form of autosomal recessive lower motor neuron disease with childhood onset**. *Am J Hum Genet* 2007, **81**:67-76.
57. Zhao GQ, Zhang Y, Hoon MA, Chandrashekar J, Erlenbach I, Ryba NJ, Zuker CS: **The receptors for mammalian sweet and umami taste**. *Cell* 2003, **115**:255-266.
